# Central nervous system (CNS) transcriptomic correlates of human immunodeficiency virus (HIV) brain RNA load in HIV-infected individuals

**DOI:** 10.1038/s41598-021-88052-7

**Published:** 2021-06-09

**Authors:** Pietro Paolo Sanna, Yu Fu, Eliezer Masliah, Celine Lefebvre, Vez Repunte-Canonigo

**Affiliations:** 1 Department of Immunology and Microbiology, The Scripps Research Institute, La Jolla, CA USA; 2 European Bioinformatics Institute (EMBL-EBI), Hinxton, UK; 3 Division of Neuroscience and Laboratory of Neurogenetics, National Institute On Aging, National Institutes of Health, Bethesda, MD USA; 4 Paris, France

**Keywords:** Diseases of the nervous system, Neural circuits

## Abstract

To generate new mechanistic hypotheses on the pathogenesis and disease progression of neuroHIV and identify novel therapeutic targets to improve neuropsychological function in people with HIV, we investigated host genes and pathway dysregulations associated with brain HIV RNA load in gene expression profiles of the frontal cortex, basal ganglia, and white matter of HIV+ patients. Pathway analyses showed that host genes correlated with HIV expression in all three brain regions were predominantly related to inflammation, neurodegeneration, and bioenergetics. HIV RNA load directly correlated particularly with inflammation genesets representative of cytokine signaling, and this was more prominent in white matter and the basal ganglia. Increases in interferon signaling were correlated with high brain HIV RNA load in the basal ganglia and the white matter although not in the frontal cortex. Brain HIV RNA load was inversely correlated with genesets that are indicative of neuronal and synaptic genes, particularly in the cortex, indicative of synaptic injury and neurodegeneration. Brain HIV RNA load was inversely correlated with genesets that are representative of oxidative phosphorylation, electron transfer, and the tricarboxylic acid cycle in all three brain regions. Mitochondrial dysfunction has been implicated in the toxicity of some antiretrovirals, and these results indicate that mitochondrial dysfunction is also associated with productive HIV infection. Genes and pathways correlated with brain HIV RNA load suggest potential therapeutic targets to ameliorate neuropsychological functioning in people living with HIV.

## Introduction

The prevalence of severe human immunodeficiency virus (HIV)-associated dementia (HAD) has decreased since the introduction of antiretrovirals, but the incidence of milder and chronic forms of HIV-associated neurocognitive disorder (HAND) and HIV-associated major depressive disorder have increased^1–10^.

HIV persists in the brain despite combination antiretroviral therapy (cART)^10–15^. HIV RNA load in the central nervous system (CNS) tends to be associated with a range of neurological manifestations^10,16–18^. Individuals with neurological symptoms but sustained serum HIV suppression often have significant cerebrospinal fluid (CSF) HIV RNA loads^16,17^. Asymptomatic CSF virus escape has also been documented by lumbar punctures^19^. Combination antiretroviral therapy decreases brain viral load, improves immunohistochemical markers of neuronal injury in primates^20^, and is associated with a reduction of neurodegeneration in humans^21^. The low CNS bioavailability of antiretrovirals (e.g., protease inhibitors, such as atazanavir) has been implicated in higher CSF HIV-1 RNA load, together with the accumulation of resistance mutations in the CNS^22^. Neurologically symptomatic CSF escape has been linked to drug resistance mutations^17,23,24^.

Despite the advent of genome-wide Omics strategies, including transcriptomic, proteomic, and metabolomic investigations, their implementation in studies of the pathogenesis of neuroHIV and the identification of druggable targets remain somewhat limited^25^. Relatively few transcriptomic studies of HIV have been conducted in human samples, although they have provided considerable insights into pathogenesis, disease progression, latency, and reactivation^26–30^.

The present study sought to identify correlations between host gene expression patterns and brain HIV RNA loads in gene expression profiles of cases in the National NeuroAIDS Tissue Consortium (NNTC), consisting of samples from three brain regions: white matter, the basal ganglia, and the frontal cortex^26,28^. We used gene set enrichment analysis (GSEA) for pathway analysis^31^ in conjunction with genesets from the Molecular Signatures Database (MSigDb), including canonical pathways in the C2 collection, including Kyoto Encyclopedia of Genes and Genomes (KEGG)^32,33^. This approach aids interpretations of genome-wide expression profiles by revealing common biological processes that are dysregulated in pathological conditions. Transcriptional analyses showed that multiple molecular systems correlated with brain HIV RNA load. Genesets that correlated with brain HIV RNA load and were concordantly dysregulated in the three brain regions studied could mostly be grouped into three broad biological processes: greater inflammation, increased transcriptional evidence of neurodegeneration, and bioenergetics dysfunction. Transcriptional evidence of neurodegeneration and bioenergetics dysfunction were also correlated with HIV RNA levels in gene expression profiles of the prefrontal cortex in a rodent model of HIV. Results also indicate that HIV expression is itself associated with mitochondrial dysfunction and that impairments in brain energetics are a central aspect of the progression and severity of neuroHIV.

## Methods

### RNA brain gene expression profiles

The gene expression dataset consisted of Affymetrix GeneChip Human Genome U133 Plus 2.0 arrays of the frontal cortex (Brodmann area 9), basal ganglia (head of the caudate nucleus), and white matter (deep frontal lobe) and was conducted by NNTC (GEO Accession No. GSE35864). A summary of the patients' clinical and demographic aspects is in Supplemental Table S1. The gene expression data were filtered and normalized as described previously^26^. Briefly, CEL files that were retrieved from GEO (Accession No. GSE35864) were analyzed using the GC Robust Multi-array Average (GCRMA) package available from Bioconductor (http://www.bioconductor.org). Normalization was implemented with the GCRMA procedure with the option fast set to FALSE in order to correctly retrieve gene–gene correlation^34^. RNA load was determined by PCR of HIV gag/pol by NNTC in each sample.

### Gene expression and pathway analysis

For pathway analysis, we used Gene Set Enrichment Analysis (GSEA), a computational method to assess whether a priori defined sets of genes show statistically significant differences between biological states^31^. GSEA was used in conjunction with genesets from the Molecular Signatures Database (MSigDb), including canonical pathways in the C2 collection^32,35^. GSEA uses the Kolmogorov–Smirnov statistical test to assess whether a predefined geneset, here a pathway from the C2 collection, is statistically enriched in differentially expressed genes by testing their distribution in the full list of genes ranked by their differential expression between two biological states^31^. The MSigDB is a comprehensive database of genesets for performing geneset enrichment analysis that represents a wide range of biological processes and diseases^32,35^. A total of 1322 canonical Pearson pathways from the MSigDB C2 collection were scored and ranked using the GSEA algorithm as described also in^12^ using R. Multiple testing adjustment was performed and significance was assessed using the False Discovery Rate (FDR) in GSEA algorithm.

### Correlation between HIV transcription and host gene and pathway expression in humans

We first computed Pearson correlations and associated *p* values between the expression levels of host genes and HIV RNA load. HIV transcription was used as a single value to estimate the overall virus expression level. Adjustments of *p* values were performed using the Benjamini–Hochberg FDR and significantly correlated host genes with HIV transcripts were selected by applying an appropriate threshold (e.g., 5% threshold by the FDR). Next, we separated the samples into two groups based on the mean expression of HIV transcripts to create groups with graded levels of HIV transcription (e.g., high and low HIV, or higher levels of expression as warranted by the results). Differential expression between groups was computed with Limma and used as an input for GSEA to identify pathways that were differentially regulated between samples with high and low HIV expression (i.e., load). Pathways that were tested were extracted from the MSigDb C2 canonical pathway collection. We selected pathways that were significantly associated with brain HIV load using the GSEA FDR < 0.05.

### Correlation between HIV transcription and host gene and pathway expression in a rodent model of HIV

HIV transgenic (Tg) rats on a mixed Wistar-Fischer background, which ensures variability of host genes and HIV expression levels, were used for the study^36^. Male HIV Tg rats, weighing between 350 and 400 g, were housed two per cage on a reverse 12 h/12 h light/dark cycle (lights off at 8:00 AM) in a temperature (20–22°C) and humidity (45–55%) controlled vivarium with ad libitum access to tap water and food pellets (PJ Noyes, Lancaster, NH, USA). All of the procedures were conducted in strict adherence to the National Institutes of Health Guide for the Care and Use of Laboratory Animals and were approved by the Institutional Animal Care and Use Committee of The Scripps Research Institute and ARRIVE guidelines (http://www.nc3rs.org.uk/page.asp?id=1357). A total of 18 gene expression profiles from prefrontal cortices of HIV Tg rats profiled by RNA-Seq as previously described^36^ were separated into two groups based on the mean expression of brain HIV transcripts to create groups with graded levels of HIV transcription (n = 7 for high brain HIV RNA load and n = 10 for low brain HIV RNA load). Differential expression between groups was computed with DESeq2 and used as an input for the GSEA to identify pathways that were differentially regulated between samples with high and low HIV expression. A normalized enrichment score and *p*-value were calculated for each pathway.

### Real-time PCR (RT-PCR)

Validation of gene expression was carried out by quantitative real-time PCR (RT-PCR) analysis in an independent set of human frontal cortex samples with either high or low HIV brain RNA loads as determined by RT-PCR (n = 5) also obtained from the NNTC. RT-PCR experiments were performed on the CFX Real-Time PCR Detection System (Bio-Rad, Hercules, CA, USA). Total RNA was reverse transcribed using the iScript cDNA Synthesis kit (Bio-Rad) with 100 ng of total RNA. Amplification was performed on a cDNA amount equivalent to 1.25 ng total RNA. Oligonucleotide primers were designed using the PrimerQuest online tool (IDT, San Diego, CA, USA). Calculations of the relative abundance of the genes under study were done by the comparative cycle threshold (Ct) method and expressed as 2-exp(ΔCt) using an empirically determined Ct of 40 as the detection threshold.

## Results

### Host transcriptional correlates of brain HIV RNA loads in human brain samples

**Figure 1:**
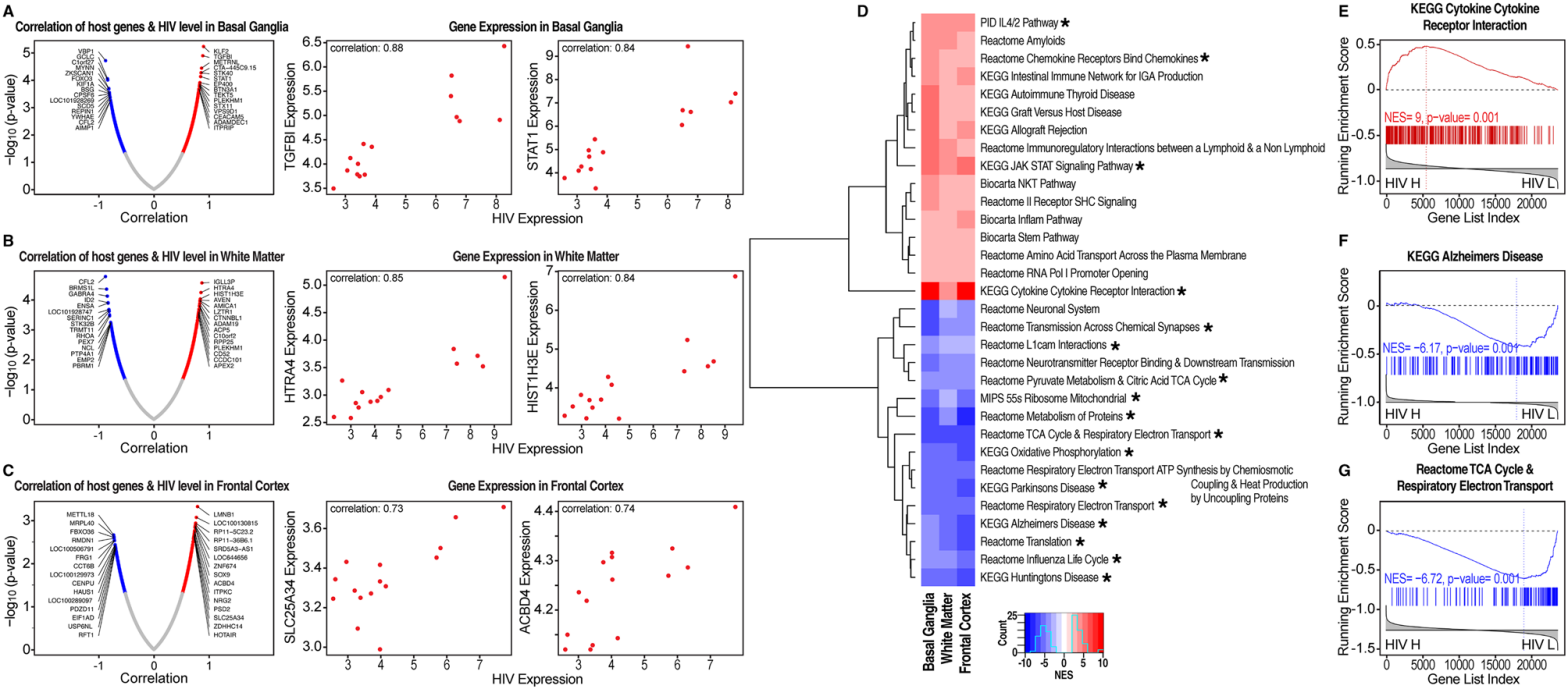
Correlation of gene expression and pathway dysregulation with brain HIV RNA load in brain regions of patients with HIV. (**A**–**C**) Host genes that correlated with brain HIV RNA load in three brain regions of patients with HIV. Volcano plots depict significant correlation with brain HIV RNA loads in the three brain regions under study (left side; also see Supplemental Table S2) and representative examples of genes (plots on the right side). (**A**) Among the top genes that correlated with HIV expression in the basal ganglia were transforming growth factor-β-induced protein (TGFBI) and signal transducer and activator of transcription 1 (STAT1). (**B**) Among the top genes that correlated with HIV expression in white matter were HtrA serine peptidase 4 (HTRA4) and histone H3.1 (HIST1H3E). (**C**) Among the top genes that correlated with HIV expression in the frontal cortex were the mitochondrial carrier protein SLC25A34 and acyl-coenzyme A binding domain containing 4 protein (ACBD4). (**D**) Gene Set Enrichment Analysis of host genes that were concordantly correlated with HIV expression in all three regions (basal ganglia [BG], white matter [WM], and frontal cortex [FC]). Asterisk indicates pathways significantly concordantly dysregulated in all three brain regions (FDR < 0.05). (**E**–**G**) Representative host pathways that were concordantly correlated with HIV expression in all three brain regions that were indicative of (**E**) increase in cytokine signaling, (**F**) neurodegeneration, and (**G**) dysregulation of bioenergetics, between samples from patients with high brain HIV RNA load (HIV-H) and samples from patients with low brain HIV RNA load (HIV-L). The example in panel (**E**) is from the basal ganglia, the one in panel F is from the frontal cortex, and the one in panel (**G**) is from white matter. Similar results were obtained for these pathways in the three brain regions as shown in (**D**). Changes in the expression of the pathway in the GSEA plots, such as the ones in (**E**–**G**), are indicated by the asymmetric distribution of genes in the geneset (vertical bars) and of the line plot of the running normalized enrichment score^31^.

Gene expression profiles from three brain regions of HIV-infected patients, including the basal ganglia, white matter, and the frontal cortex, were used for the study. The gene expression data were filtered and normalized as described previously^26^. Pearson correlations and associated *p* values were computed between the expression levels of host genes and HIV transcripts for each brain region as outlined in the Methods section. Genes with a high degree of correlation with brain HIV RNA loads in each of the three brain regions studied are shown in Fig. 1A–C and Supplemental Table S2.

We applied GSEA, a computational method that assesses whether a priori-defined genesets show statistically significant differences between biological states^31^, to compare gene expression profiles associated with high brain HIV RNA loads. GSEA was used in conjunction with genesets from the MSigDb, including canonical pathways in the C2 collection^32^. In total, we identified 254 pathways in the basal ganglia, 104 pathways in white matter, and 393 pathways in the frontal cortex that were significantly enriched/decreased in samples with high brain HIV load (FDR < 0.05).

The top differentially regulated pathways in the three brain regions (concordantly changed in all regions) ranked by the normalized enrichment score (NES) are shown in Fig. 1D. Overall, the pathway analysis indicated that host genesets correlated with brain HIV RNA load and were concordantly dysregulated in the three brain regions studied, which could mostly be grouped into three broad biological processes: inflammation, neurodegeneration, and bioenergetics. Pathways related to inflammation, neurodegeneration, and bioenergetics were found to be correlated with HIV levels in all three brain regions (Fig. 1D–G). Pathways that are representative of inflammation and were concordantly dysregulated across brain regions were primarily representative of increased cytokine signaling and directly correlated with brain HIV RNA load. Pathways that are representative of neurodegeneration included pathways that are representative of gene expression changes in Alzheimer’s disease (AD), Parkinson’s disease (PD), and Huntington’s disease (HD) were found to be dysregulated across all brain regions.

**Figure 2:**
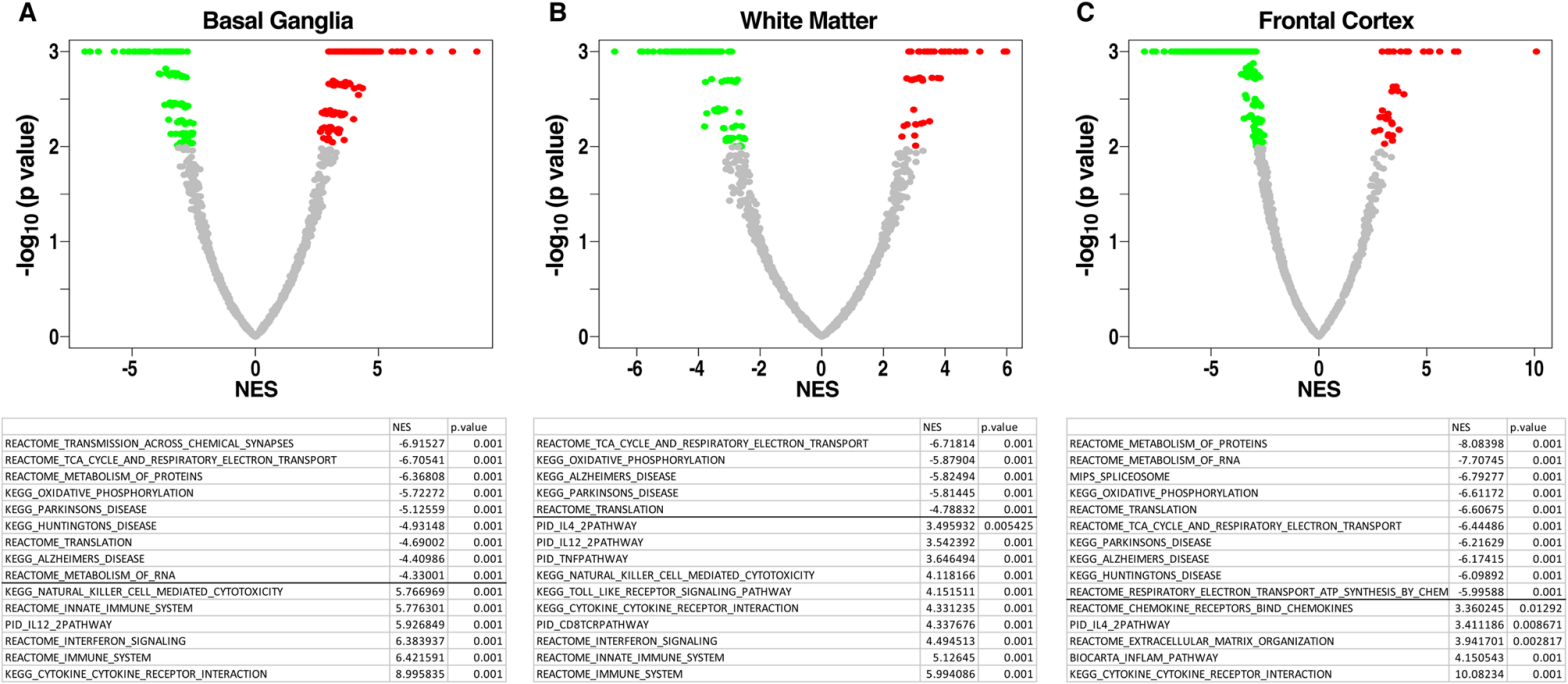
Correlation between pathway dysregulation and brain HIV RNA load in three brain regions of patients with HIV. (**A**–**C**) Scatter (volcano) plots that depict differentially regulated pathways by GSEA between high brain HIV RNA load (HIV H) and low brain HIV RNA load (HIV L) in (**A**) the basal ganglia, (**B**) white matter, and (**C**) the frontal cortex. NES, running normalized enrichment score. Tables for each brain region show representative downregulated and upregulated pathways. Pathways with substantial overlap are omitted from these lists. Complete lists are shown in Supplementary Tables S3-6.

Pathways that are representative of bioenergetics, including pathways that are related to oxidative phosphorylation, electron transfer, and the tricarboxylic acid (TCA) cycle, were inversely correlated in all three brain regions studied (Fig. 2). Transcripts of several genes involved in energy metabolism were inversely correlated with HIV RNA load in all three brain regions, including α-enolase (ENO1), a glycolytic enzyme that catalyzes the conversion of 2-phosphoglycerate to phosphoenolpyruvate, glyceraldehyde 3-phosphate dehydrogenase (GAPDH), which catalyzes the sixth step in glycolysis, converting glyceraldehyde 3-phosphate to d-glycerate 1,3-bisphosphate, and the mitochondrial membrane ATP synthase subunit C locus 3 (ATP5G3), Supplemental Table S2.

**Figure 3:**
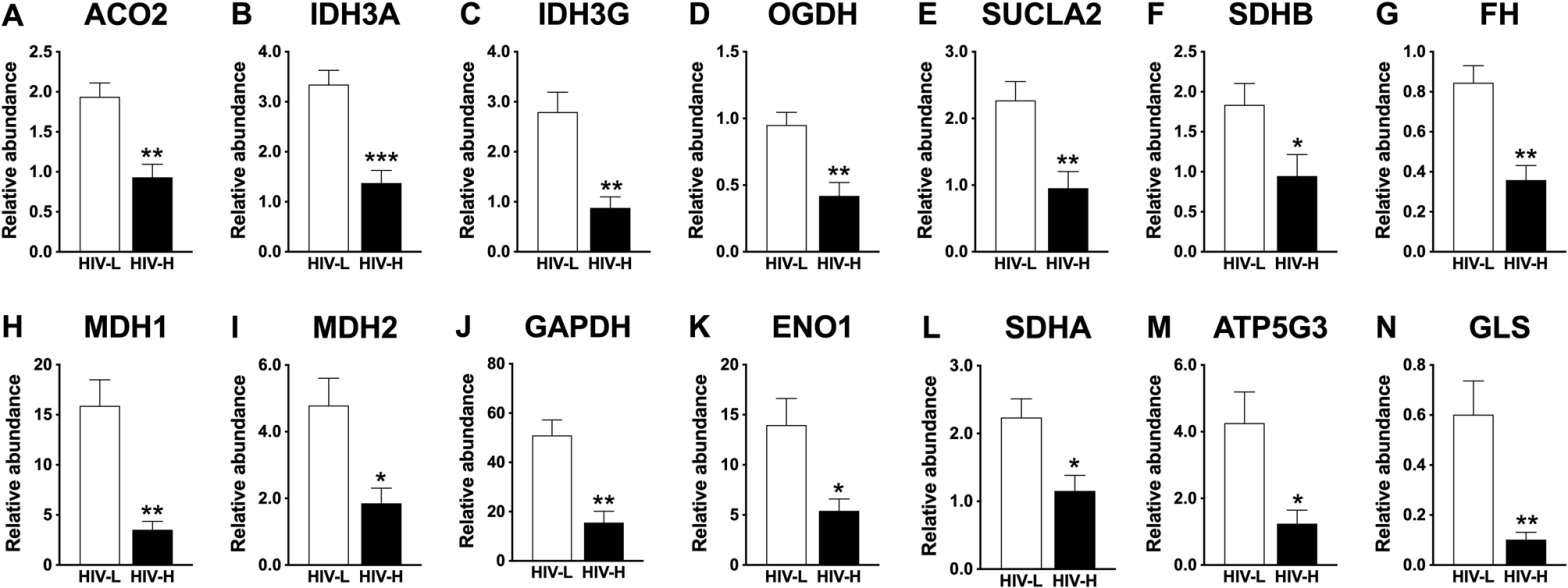
Dysregulation of the expression of genes related to energy metabolism and the tricarboxylic acid (TCA) cycle in patients with high brain HIV RNA load. The mRNA levels of genes related to energy metabolism and the TCA cycle were compared in an independent sample of frontal cortices of patients with low brain HIV RNA load (HIV L) and high brain HIV RNA load (HIV H) by RT-PCR (n = 5). Reduced levels were observed for the mRNAs of mitochondrial TCA cycle enzymes, including (**A**) aconitase 2 (ACO2), (**B**) isocitrate dehydrogenase subunits α (IDH3A) and (**C**) γ (IDH3G), (**D**) α-ketoglutarate dehydrogenase (2-oxoglutarate dehydrogenase E1 component, OGDH), (**E**) succinyl-CoA ligase subunit β (SUCLA2), (**F**) succinate dehydrogenase (SDHB) subunit β, (**G**) fumarate hydratase (FH), and (**H**) malate dehydrogenase 1 (MDH1) and 2 (MDH2) **(I)** as well as for the glycolytic enzymes (**J**) glyceraldehyde 3-phosphate dehydrogenase (GAPDH) and (**K**) α-enolase (enolase 1 [ENO1]) and mitochondrial respiratory chain proteins (**L**) succinate dehydrogenase complex, subunit A (SDHA) and (**M**) mitochondrial membrane ATP synthase subunit C locus 3 (ATP5G3) and for the mRNA of (**N**) mitochondrial glutaminase (GLS). *p < 0.05, **p < 0.01, ***p < 0.001.

The differential expression of genes involved in energy metabolism and the TCA cycle was confirmed in an independent set of frontal cortices from individuals with high and low brain HIV RNA loads by quantitative real time PCR (RT-PCR), Fig. 3.

Pathway dysregulations in each of the three brain regions studied are reviewed next.

#### Basal ganglia

**Figure 4:**
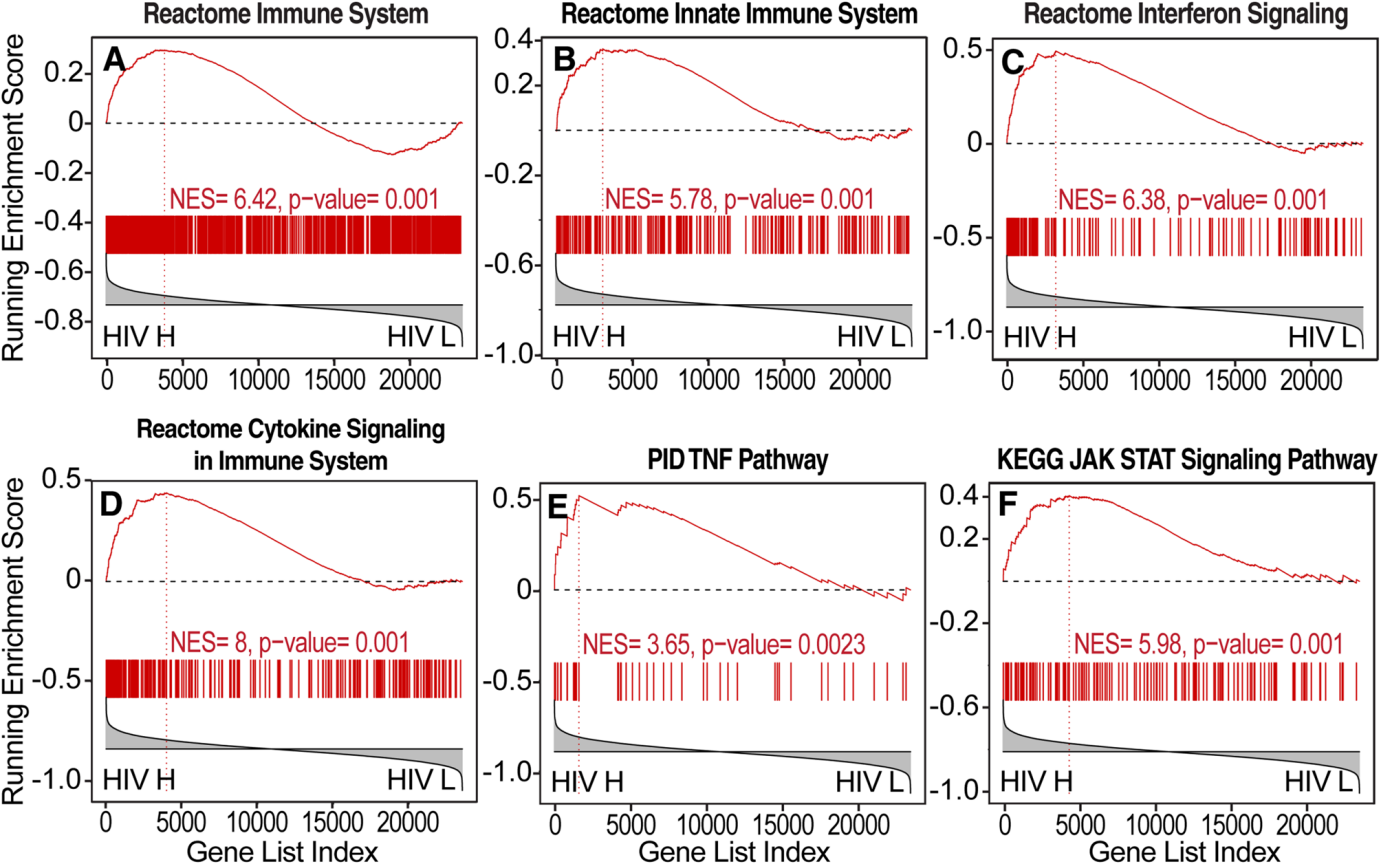
Increase in neuroinflammation correlated with brain HIV RNA load in the basal ganglia. (**A**, **B**) Pathway analysis by GSEA provided extensive evidence of immune activation and inflammation, including (**C**) increases in interferon signaling, (**D**) cytokine signaling, (**E**) tumor necrosis factor α (TNF-α) signaling, and (**F**) JAK STAT pathway activation. HIV H, high brain HIV RNA load; HIV L, low brain HIV RNA load.

**Figure 5:**
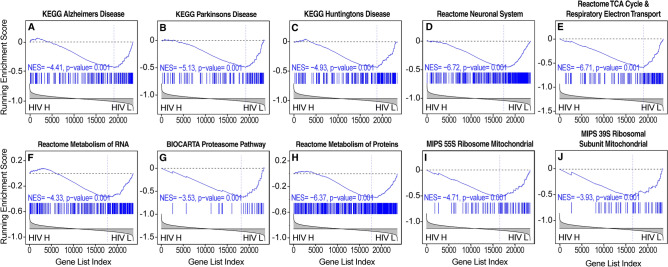
Evidence of increase in neurodegeneration and impairments in bioenergetics correlated with brain HIV RNA load in the basal ganglia. (**A**–**C**) Transcriptional changes that are indicative of neurodegeneration, including pathways that are representative of gene expression changes in (**A**) Alzheimer’s disease (AD), (**B**) Parkinson’s disease (PD), and (**C**) Huntington’s disease (HD), were associated with high brain HIV RNA load. (**D**) Gene expression changes indicated that reductions of neuronal signaling that are indicative of a reduction of trophism and synaptodendritic injury were associated with high brain HIV RNA load. (**E**) Transcriptional evidence of impairments in mitochondrial function and energy metabolism were also correlated with brain HIV RNA load in the basal ganglia as well as evidence of impairments in (**F**) RNA metabolism, (**G**, **H**) proteostasis, and (**I**, **J**) lower mitochondrial protein synthesis. HIV H, high brain HIV RNA load; HIV L, low brain HIV RNA load.

The pathways that showed the highest correlation with brain HIV load in the basal ganglia included pathways indicative of inflammation and immune system activation, including interferon type I and II and cytokine signaling, Toll-like receptor signaling, tumor necrosis factor-α signaling, JAK-STAT signaling, and interferon (Figs. 2 and 4). Inversely correlated with brain HIV RNA load in the basal ganglia were several pathways that are indicative of neural degeneration, including pathways that are representative of gene expression changes in AD, PD, and HD (Figs. 2 and 5), indicative of a reduction of neuronal signaling and trophism. Also inversely correlated with brain HIV RNA load were pathways related to synaptic plasticity, α-amino-3-hydroxy-5-methyl-4-isoxazolepropionic acid (AMPA) receptor trafficking, and axonal function (Figs. 2 and 5, Supplemental Table S3). Pathways that are related to energy metabolism, including the TCA cycle, oxidative phosphorylation, and glycolysis, were concomitantly reduced with higher virus brain RNA load (Figs. 2 and 5). Transcriptional evidence of a catabolic milieu and evidence of impairments in proteostasis and mitochondrial protein synthesis were also associated with higher brain HIV RNA load (Figs. 2 and 5). Among the genes that showed higher correlations with virus titers were transforming growth factor, β-induced (TGFBI), signal transducer and activator of transcription 1 (STAT1), meteorin-like (METRNL), interferon-induced with helicase C domain 1 (IFIH1), interferon-inducible protein 16 (IFI16), IFI30, interferon-inducible transmembrane 1 (IFITM1), and γ-interferon-inducible lysosomal thiol reductase (GILT; Fig. 1A, Supplemental Table S4)*.*

#### White matter

**Figure 6:**
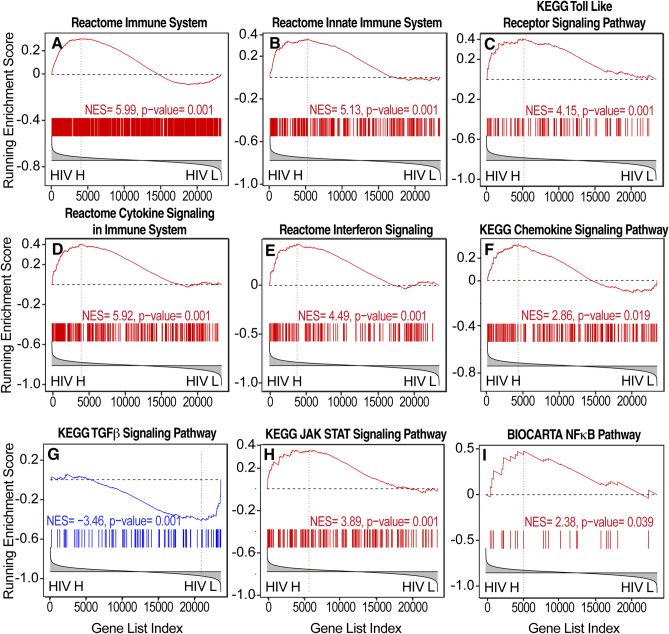
Evidence of increase in neuroinflammation correlated with brain HIV RNA load in white matter. (**A**–**C**) The pathway analysis by GSEA demonstrated broad immune activation and inflammation, characterized by increases in (**D**) cytokine, (**E**) interferon, and (**F**) chemokine signaling, (**G**) a decrease in transforming growth factor β (TGF-β) pathway signaling, (**H**) an increase in JAK STAT pathway signaling, and (**I**) an increase in nuclear factor κB (NFκB) pathway signaling. HIV H, high brain HIV RNA load; HIV L, low brain HIV RNA load.

**Figure 7:**
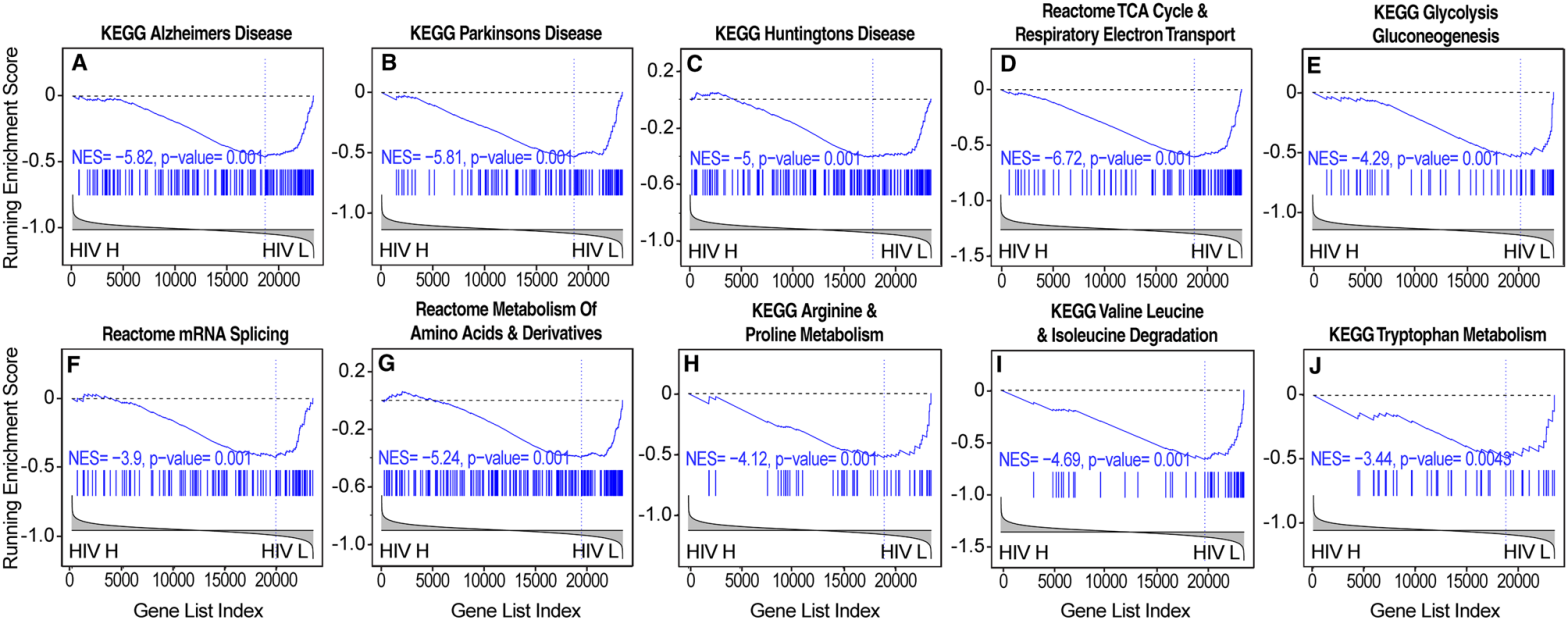
Evidence of increase in neurodegeneration and impairments in bioenergetics correlated with brain HIV RNA load in white matter. (**A**–**C**) The pathway analysis by GSEA demonstrated transcriptional changes that are indicative of neurodegeneration and included pathways representative of gene expression changes in AD, PD, and HD. (**D**) Gene expression changes indicative of a reduction of energy metabolism, including  reduced gene expression of the TCA cycle, electron transport, (**E**) reduced expression of glycolytic genes; (**F**) reduced expression of RNA processing genes, and (**G**–**J**) reduced expression of amino acid metabolic pathways**.** HIV H, high brain HIV RNA load; HIV L, low brain HIV RNA load.

Transcriptional evidence of immune activation and inflammation in white matter that were positively correlated with brain HIV RNA load included pathways indicative of increases in cytokines, interferons, and chemokines, including increases in JAK STAT and nuclear factor κB (NFκB) signaling, interferon signaling, and inflammasome activation (Figs. 2 and 6, Supplemental Table S5). Transcriptional evidence of increased neurodegeneration and impaired bioenergetics was also correlated with brain HIV RNA load in white matter, including pathways representative of AD, PD, and HD, reductions of TCA cycle and electron transport, and glycolytic genes (Figs. 2 and 7). Concomitant evidence of a catabolic milieu was also associated with higher brain HIV RNA load (Figs. 2 and 7).

#### Frontal cortex

**Figure 8:**
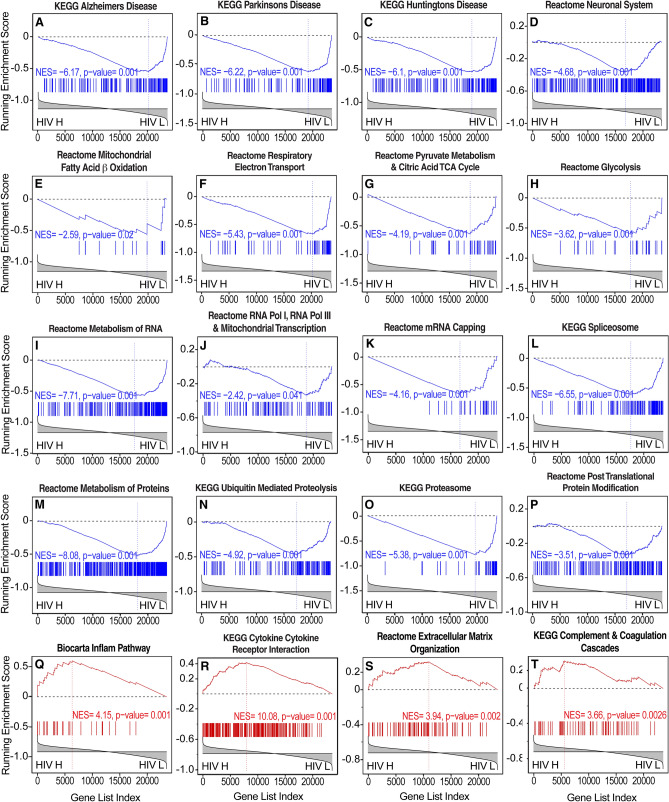
Representative pathways indicative of neurodegeneration, impairments in bioenergetics, and neuroinflammation correlated with brain HIV RNA load in the frontal cortex. (**A**–**C**) The pathway analysis by GSEA demonstrated transcriptional changes that are indicative of neurodegeneration, including pathways representative of gene expression changes in AD, PD, and HD.  (**D**) Gene expression changes indicated a reduction of neuronal signaling that is consistent with synaptodendritic injury, (**E**–**H**) impairments in mitochondrial function and energy metabolism, including reductions of TCA cycle, electron transport, and glycolytic genes, (**I**–**L**) impairments in RNA synthesis and processing, (**M**–**P**) proteostasis and protein synthesis, (**Q**, **R**) increased inflammation and cytokine signaling, (**S**) extracellular matrix, and (**T**) complement genes.  HIV H, high brain HIV RNA load; HIV L, low brain HIV RNA load.

Pathways reflecting changes in the expression of neuronal genes, including axonal and synaptic transcripts, trophic signaling, and mitochondrial and other key genes that are associated with neurodegenerative conditions were dysregulated in the frontal cortex (Figs. 2 and 8). Like the basal ganglia and white matter, these included genesets involved in AD, PD, and HD and evidence of reductions of synaptodendritic injury, synaptic signaling and plasticity, neurotrophin signaling, and energy metabolism (Fig. 7). The latter included reductions of genesets that are representative of TCA cycle, electron transport, and glycolytic genes (Figs. 2, 3 and 8). A catabolic milieu was also indicated by impairments in RNA synthesis and processing and impairments in proteostasis and protein synthesis that are associated with brain HIV RNA load (Fig. 8). Immune activation and inflammation were also correlated with brain HIV RNA load (Fig. 8). Pathways representative of increased inflammation and cytokine signaling were also correlated with brain HIV RNA load in the frontal cortex (Fig. 8).

### Transcriptional correlates of brain HIV RNA load in a rodent model

**Figure 9:**
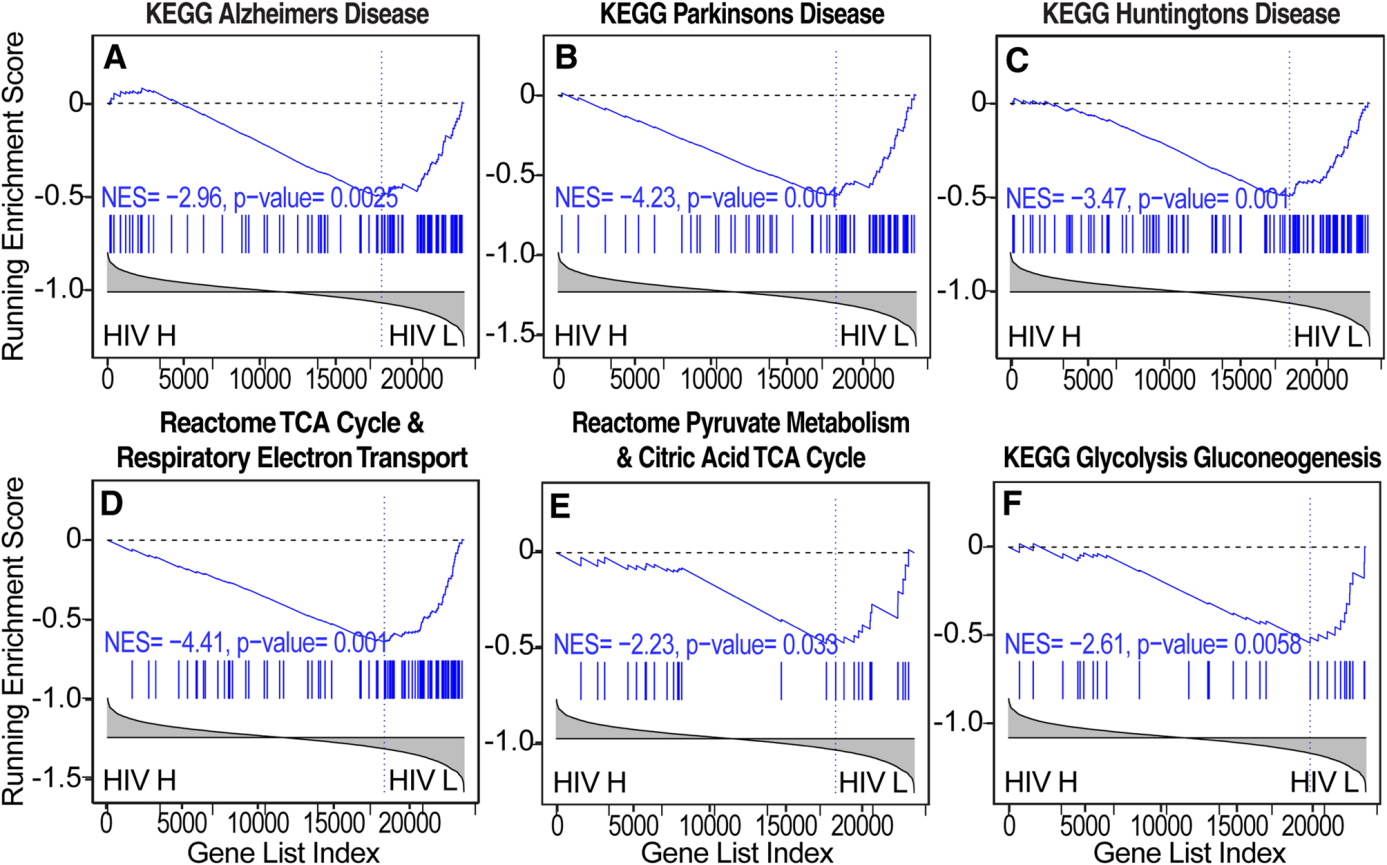
Representative pathways indicative of neurodegeneration and impairments in bioenergetics correlated with brain HIV RNA load in the prefrontal cortex in a rodent model of HIV. (**A**–**C**) The pathway analysis by GSEA showed transcriptional changes that are indicative of neurodegeneration-like changes, including pathways that are representative of gene expression changes in AD, PD, and HD, and (**D**–**F**) gene expression changes that are indicative of a reduction of energy metabolism, including reductions of TCA cycle, electron transport, and glycolytic genes. HIV H, high brain HIV RNA load; HIV L, low brain HIV RNA load.

HIV transgenic rats express multiple HIV products in disease-relevant cells in the brain, such as microglia and astrocytes, under control of the viral LTR promoter^37–39^. These rats also have similar brain gene expression changes as humans with HIV^26,36,38^. The repertoire of pathway dysregulations in humans with HIV in the present study was generally consistent with observations in HIV transgenic rats^36,38^. As depicted in Fig. 9, using previously reported gene expression profiles of the frontal cortex in HIV transgenic rats on a mixed genetic background^36^, the expression of genesets representative of neurodegeneration and bioenergetics, although not inflammation-related genesets, was significantly correlated with brain HIV RNA loads in the frontal cortex in HIV transgenic rats, despite a lower HIV RNA dynamic range (Fig. 9).

## Discussion

The molecular mechanisms of HIV-associated neurotoxicity have been only partially elucidated. cART has itself been implicated in neurotoxicity^40–42^. Brain HIV viral load and indirect indicators, such as CSF HIV RNA load, are generally, albeit variably, inversely correlated with neuropsychological scores^10,43–45^. To better highlight genes and pathway dysregulations that have greater potential relevance to neuroHIV severity and progression, we correlated transcriptional alterations of functional pathways and genes in three brain regions in human cases of HIV with brain HIV RNA load. We used GSEA, a knowledge-based computational strategy that interrogates a genome-wide expression profile dataset using a priori-defined genesets of functionally related genes^46^. GSEA was used in conjunction with the MSigDb, including canonical pathways in the C2 collection^32^.

Here, we found that transcriptional evidence of inflammation was more pronounced in white matter and the basal ganglia. Pathways that are representative of cytokine signaling were correlated with brain HIV RNA load in all three brain regions, consistent with the contribution of inflammatory processes to cortical, subcortical, and white matter injury^47^. Increases in interferon signaling were associated with high brain HIV RNA load in the basal ganglia and white matter. However, despite considerable activation of interferon-related pathways in the frontal cortex of HIV cases versus uninfected control individuals in the present human neuroHIV dataset^26^, their overall expression was not significantly correlated with brain HIV RNA load in the frontal cortex. In fact, significant activation of interferon signaling is seen in the frontal cortex even in the absence of neurocognitive impairment^26^. Conversely, the present results highlight that in subcortical regions such as the basal ganglia and white matter, the activation of interferon signaling was directly correlated with brain HIV RNA load.

Effective cART is associated with lower intrathecal inflammation^48–50^. A multilevel analysis of the neuropathogenesis of neurocognitive impairment in HIV showed that brain HIV RNA load was significantly correlated with inflammation and markers of neurodegeneration^45^. Significant correlations were also found between inflammatory markers, particularly type I interferon, and brain or CSF HIV RNA load has been observed in both recent cohorts^10,51–53^ and earlier ones^54–56^.

In the present study, brain HIV RNA load was inversely correlated with genesets that are indicative of neuronal and synaptic genes, particularly in the cortex, indicative of synaptic injury and neurodegeneration. Genesets that are related to neurodegeneration and were found to be dysregulated in cases with high brain HIV RNA load included pathways that are representative of gene expression changes in AD, PD, and HD. Established immunohistochemical markers of neuropathological changes that underlie the progression of neuroHIV include pre- and postsynaptic markers, such as synaptophysin (SYP) and microtubule-associated protein-2 (MAP2)^45,57^, and abnormal protein aggregates, such as β-amyloid^58–62^. MAP2 and SYP are indicators of synaptic integrity, and their expression in the frontal cortex is inversely correlated with plasma and brain HIV RNA load^45^. β-amyloid plaque burden in the frontal cortex also correlated with brain HIV RNA load, and SYP and β-amyloid were negatively correlated with HIV RNA load^45^. Transcriptional evidence of alterations of proteostasis that were associated with brain HIV RNA load was also found, particularly in the basal ganglia and frontal cortex. Altogether, these results indicate that brain HIV RNA load is correlated with synaptodendritic injury and dysfunctional protein clearance. Evidence of neurodegeneration was also found in the basal ganglia and white matter, in addition to the frontal cortex. This is consistent with mounting evidence of the involvement of the basal ganglia and white matter in aging and neurodegenerative diseases, such as AD and PD^63–71^.

Emerging neuropathology concepts in neuroHIV also suggest a degree of pathogenic similarity and possibly overlap between neuroHIV and AD. Several studies reported increases in amyloid deposition in older HIV-infected individuals, suggesting that long-term HIV and antiretroviral therapy might interfere with the clearance of proteins, such as amyloid-β peptide, and worsen neuronal damage and cognitive impairment in this population^58,60,61,72, 73^. In one study, amyloid-β plaques in HIV+ brains, although immunohistologically different from those in symptomatic AD brains, were associated with HAND among apolipoprotein E (APOE) ε4 carriers, suggesting the possibility of convergent mechanisms of pathogenesis^61^. In vitro studies indicate that HIV proteins can disrupt different steps of amyloid pathways^59,74–76^. The HIV protein Tat, which is produced by the HIV reservoir in the brain even in the setting of the suppression of viral replication^77^, has been shown to bind amyloid-β peptide, promoting amyloid aggregation and neurotoxicity^78^. When Tat was injected in the brains of amyloid precursor protein and presenilin-1 transgenic mice (APP-PS1), it colocalized with APP^78^. Furthermore, crossing Tat transgenic mice with APP-PS1 transgenic mice resulted in an increase in amyloid-β deposition, neurodegeneration, neuronal apoptotic signaling, and phosphorylated Tau compared with PSAPP mice^79^, indicating that Tat likely contributes to AD-like pathology in HIV. As the HIV-positive population ages, individuals with both HIV and canonical AD pathology are beginning to be diagnosed. A recent case report of AD in an HIV-infected patient who was positive for amyloid on positron emission tomography^80^ was followed by approximately 20 more cases^81^. Thus, current issues of debate include the possibility of HAND/AD mixed dementia and the hypothesis that HIV may represent a predisposing factor for AD^80–83^. Supporting the latter possibility, combined CSF biomarker risk for AD in an Australian HIV-positive cohort was found to be more than 10-times greater than in age-matched controls^82^.

The present data also show that the gene expression evidence of impairments in bioenergetics are associated with brain HIV RNA load and are correlated with transcriptional evidence of inflammation and neurodegeneration. Mitochondrial dysfunction has been implicated in neurodegenerative disease (e.g., AD^84–86^ and PD^86,87^), aging^88^, and neuroHIV, particularly in adverse ageing in older people who live with HIV^89^. Frontal cortices from patients with HAND showed mitochondrial abnormalities, including a reduction of mitochondrial biogenesis^90^. The accumulation of mutations and deletions of mitochondrial DNA (mtDNA)^91,92^ altered mitochondrial fission and fusion^93^. Experimental evidence shows that HIV products, such as Tat and gp120, can cause mitochondrial damage^94–100^. Mitochondrial dysfunction is also implicated in the toxicity of some antiretrovirals^101^. The present data indicate that mitochondrial dysfunction is a key component of the neuropathogenesis of HIV infection itself.

In the present study, we found that a rodent model of HIV, in which several HIV products are expressed in disease-relevant glial cells (e.g., microglia and astrocytes) but not in neurons^37,38^, exhibited dysregulations of bioenergetics- and neurodegeneration-related genesets that were associated with brain HIV RNA paralleled findings in gene expression profiles of patients with HIV. In agreement with the present results, mitochondria from this HIV rodent model were found to have significantly lower oxygen consumption rates, basal respiration, ATP production, maximal respiratory capacity, spare capacity, proton leakage, and non-mitochondrial respiration^96^.

Emphasis has been rightfully placed on impairments in oxidative phosphorylation in neuroHIV and degenerative diseases, such as AD^84,102–105^. The present pathway analysis underscores the potential contribution of disruptions of the TCA cycle and β-oxidation in the mitochondrial matrix, in which oxidative phosphorylation depends on the supply of reducing equivalents from the end-oxidation of nutrients^106^. The TCA cycle and oxidative phosphorylation are tightly coupled. The TCA cycle produces the reducing equivalents NADH and FADH2, which are required for electron transfer through the mitochondrial respiratory chain or electron transport chain (ETC). The oxidation of NADH and FADH2 in complexes I and II of the ETC is required to maintain the function of the TCA cycle^106^. As electrons are transported through the complexes, the ETC produces a mitochondrial membrane potential that generates ATP. Mitochondrial complexes I and II in the ETC replenish NAD+ and FAD, respectively, allowing the oxidative TCA cycle to function^106^. Thus, lower expression of TCA cycle-related genes may play a crucial role in impairments in mitochondrial oxidative phosphorylation in neuroHIV. Tricarboxylic acid cycle metabolites are also important for the biosynthesis of nucleotides, lipids, and proteins^106^. Mounting evidence indicates that metabolites in the TCA cycle are also involved in controlling chromatin modifications, DNA methylation, and post-translational modifications of proteins^106^. Acetyl-coenzyme A levels regulate chromatin dynamics by providing acetyl groups for the acetylation of histones by histone acetyltransferases^107–110^. The TCA cycle predominates in neurons, whereas glycolysis predominates in astrocytes^111^. Thus, decreases in TCA cycle-related gene expression in neuroHIV may be a correlate of lower neuronal energy metabolism. Evidence of alterations of TCA cycle capacity and expression that are induced by HIV infection in the scientific literature is limited. Interestingly, CSF metabolomics showed that worsening cognitive status in HIV-infected patients is associated with the accumulation of citrate and acetate, which are suggestive of disruptions of TCA cycle function^112^. A separate CSF metabolomic analysis of young individuals with HIV who were on ART found a dysregulation of TCA cycle intermediates, such as succinate and malate, among other metabolites, differed when compared with HIV-negative controls^113^. Relevant to the present results, we recently found that escalated (dependent) methamphetamine self-administration in HIV transgenic rats was associated with the lower expression of TCA cycle-related genes in conjunction with transcriptional evidence of increases in inflammation and neurodegeneration^36^.

Selected genes that encode enzymes that are involved in glycolysis, such as ENO1 and GAPDH, were concordantly dysregulated in all three brain regions that were studied herein, and the expression of genesets that are representative of glycolysis was reduced particularly in the basal ganglia and frontal cortex, thus indicating that alterations of glycolysis and reductions of the TCA cycle and electron transport are key bioenergetic alterations that are correlated with brain HIV RNA load.

In conclusion, we explored functional pathways that are associated with brain HIV load as a correlate of neuroHIV disease severity and progression by the pathway analyses of transcriptional profiles from brain regions of humans with HIV. We found that brain HIV RNA load correlated with transcriptional evidence of an increase in inflammation. Evidence of increased cytokine signaling was associated with high HIV RNA across the three brain regions studied. Increases in interferon signaling were correlated with high brain HIV RNA load in the basal ganglia and white matter although not in the frontal cortex. We also found transcriptional evidence of neurodegeneration that involved pathways that reflect changes in axonal and synaptic genes and trophic signaling, among others. Lastly, we found transcriptional evidence of impairments in bioenergetics that involved oxidative phosphorylation and the TCA cycle across all three brain regions. Transcriptional evidence of mitochondrial dysfunction was also associated with brain HIV RNA load and correlated with transcriptional evidence of inflammation and neurodegeneration, indicating that mitochondrial dysfunction is a key component of the neuropathogenesis of HIV infection itself.

## Supplementary Information

Supplementary Information 1.

Supplementary Information 2.

Supplementary Information 3.

Supplementary Information 4.

Supplementary Information 5.

Supplementary Information 6.

Supplementary Information 7.
