# Supplementary material for: Central nervous system (CNS) transcriptomic correlates of human immunodeficiency virus (HIV) brain RNA load in HIV-infected individuals
Source: Sci Rep. 2021 Jun 9;11:12176. doi: 10.1038/s41598-021-88052-7 (PMC8190104; doi:10.1038/s41598-021-88052-7)
Supplement: Supplementary file 1 — Supplementary Information 1. [file 41598_2021_88052_MOESM1_ESM.docx]

**Supporting Information**

**Central nervous system (CNS) transcriptomic correlates of­­­ human immunodeficiency virus (HIV) brain RNA load in HIV-infected individuals**

Pietro Paolo Sanna,^1^* Yu Fu,^1, 2^ Eliezer Masliah,^3^ Celine Lefebvre,^1, 4^

and Vez Repunte-Canonigo^1^*

**^1^**Department of Immunology and Microbiology, The Scripps Research Institute, La Jolla, CA, USA.

**^2^**European Bioinformatics Institute (EMBL-EBI), Hinxton, United Kingdom.

**^3^**Division of Neuroscience and Laboratory of Neurogenetics, National Institute on Aging, National Institutes of Health, Bethesda, MD, USA.

**^4^**Paris, France.

Running title: Gene expression correlates of HIV brain RNA load.

Keywords: Alzheimer’s disease, Parkinson’s disease, Krebs cycle, glycolysis, dementia, cognitive impairment.

*Correspondence:
[psanna@scripps.edu](mailto:psanna@scripps.edu)

[canonigo@scripps.edu](mailto:canonigo@scripps.edu)

**Supplemental Tables:**

**Table S1. Clinical and demographic aspects.**

**Table S2. Genes concordantly correlated with brain HIV RNA loads across brain regions.**

**Table S3. Pathways correlated with HIV RNA load in the Basal ganglia.**

**Table S4. Pathways correlated with HIV RNA load in the Frontal Cortex.**

**Table S5. Pathways correlated with HIV RNA load in the White Matter.**

**Table S6. Pathways correlated with HIV RNA load in the rat Frontal Cortex.**
